# Use of reproductive health services among women using long- or short-acting contraceptive methods – a register-based cohort study from Finland

**DOI:** 10.1186/s12889-022-13581-3

**Published:** 2022-06-14

**Authors:** Tuire Helene Saloranta, Frida Katrin Gyllenberg, Anna But, Mika Gissler, Oskari Heikinheimo, Merja Kristiina Laine

**Affiliations:** 1 Myyrmäki Health Center, Jönsaksentie 4, 01600 Vantaa, Finland; 2 Department of General Practice and Primary Care, University of Helsinki, Tukholmankatu 8 B, 00029 HUS Helsinki, Finland; 3 Department of Obstetrics and Gynecology, University of Helsinki, Haartmaninkatu 2, 00029 HUS Helsinki, Finland; 4 Department of Public Health, University of Helsinki and Helsinki University Hospital, Tukholmankatu 8 B, 00029 HUS Helsinki, Finland; 5 Department of Knowledge Brokers, Finnish Institute for Health and Welfare, Mannerheimintie 166, 00271 Helsinki, Finland; 6 Research Centre for Child Psychiatry, University of Turku, Lemminkäisenkatu 3 (Teutori 3. krs), 20014 Turku, Finland; 7 Region Stockholm, Academic Primary Health Care Centre, Solnavägen 1 E, 113 65 Stockholm, Sweden; 8 Department of Molecular Medicine and Surgery, Karolinska Institute, Solnavägen 1, 17177 Stockholm, Sweden; 9 Department of Obstetrics and Gynecology, University of Helsinki and Helsinki University Hospital, PO Box 140, FI-00029 HUS Helsinki, Finland; 10 Folkhälsan Research Center, Topeliuksenkatu 20, 00250 Helsinki, Finland

**Keywords:** Long-acting reversible contraception, Short-acting reversible contraception, Reproductive health services

## Abstract

**Background:**

Long-acting reversible contraceptives (LARCs) have superior contraceptive efficacy compared to short-acting reversible contraceptives (SARCs) and choosing LARCs over SARC methods reduces the need for abortion care. However, little is known how initiating these methods associates with the subsequent overall need of reproductive health services including family planning services, and visits for gynecological reasons in primary and specialized care.

**Methods:**

We followed altogether 5839 non-sterilized women aged 15–44 years initiating free-of-charge LARC methods (*n* = 1689), initiating or switching SARC methods (*n* = 1524), or continuing with the same SARC method (*n* = 2626) at primary care family planning clinics in the City of Vantaa, Finland, 2013–2014 for 2 years using Finnish national health registers.

We assessed the use of reproductive health services, namely attending public primary or specialized health care for gynecological reasons or attending the family planning clinics by applying unadjusted and adjusted negative binomial regression models on visit counts.

**Results:**

A total of 11,290 visits accumulated during the two-year follow-up: 7260 (64.3%) at family planning clinics, 3385 (30.0%) for gynecological reasons in primary, and 645 (5.7%) in specialized health care. Altogether 3804 (52.4%) visits at the family planning clinics were for routine checkup, and 3456 (47.6%) for other reasons. Women initiating LARC methods used reproductive health services for reasons other than routine checkups similarly as women initiating or switching SARC methods (adjusted incidence rate ratio 0.93, 95% CI 0.82–1.05), while women continuing with SARC methods used the services less frequently (0.65, 0.59–0.72). Women initiating free-of-charge LARC and those continuing with the same SARC method used services less for abortion care than women initiating or switching SARC (adjusted incidence rate ratios 0.05, 95% CI 0.03–0.08 and 0.16, 95% CI 0.11–0.24, respectively).

**Conclusions:**

While women initiating LARC methods have lower need for abortion care compared to women initiating SARC methods, women initiating both LARC and SARC methods have similar overall need for reproductive health services. In contrast, women continuing with their SARC method need reproductive health services less than women initiating LARC or a new SARC method. These service needs should be acknowledged when planning and organizing family planning services, and when promoting long-acting reversible contraception.

**Supplementary Information:**

The online version contains supplementary material available at 10.1186/s12889-022-13581-3.

## Background

Long-acting reversible contraceptives (LARCs; including intrauterine devices and contraceptive implants) are highly effective and actively promoted as first-line contraceptives for all women even with free-of-charge programs to reduce the financial barrier for initiation [1, 2]. As these methods are well tolerated and highly continued, significantly reduce the need for induced abortion and do not require regular prescription or provision, [3–5] it may be assumed that women initiating LARC methods need reproductive health services, such as family planning services on method related issues, or primary or specialized health care services on gynecological issues, less than those using short-acting reversible contraception (SARC; including pills, patches and rings).

Whilst the efficacy, health benefits, possible adverse events, and discontinuation of LARC methods have been well studied, knowledge is scarce regarding the overall use of reproductive health services by women using LARC methods compared to women choosing SARC methods. One study in the US showed that women choosing LARC methods had less overall Medicaid healthcare costs than women choosing SARC, but the study did not focus on costs due to reproductive health care, neither is Medicaid likely to be comparable to the Finnish public health care, where visits concerning contraception are free of charge and the out of pocket costs of visits in primary care (16€,~ 14£) and specialized care (30€,~ 26£) are low [6]. In Finland, private service providers offer health services alongside the public healthcare system, but with substantially higher cost, only partially covered by the National Health Insurance.

Understanding the overall need for services is vital for ensuring sufficient contraceptive services, and to be able to estimate the overall cost-benefit ratio of free-of-charge LARC programs.

In this study, we aimed to assess the use of reproductive health services among free-of-charge LARC initiators compared to women initiating or switching between SARC methods or continuing with the same SARC method within a public program providing all women their first LARC method free of charge. Our main aim was to assess potential differences in the use of reproductive health services between these three groups when accounting for confounding that inevitably arise in real-life settings.

## Methods

**Fig. 1:**
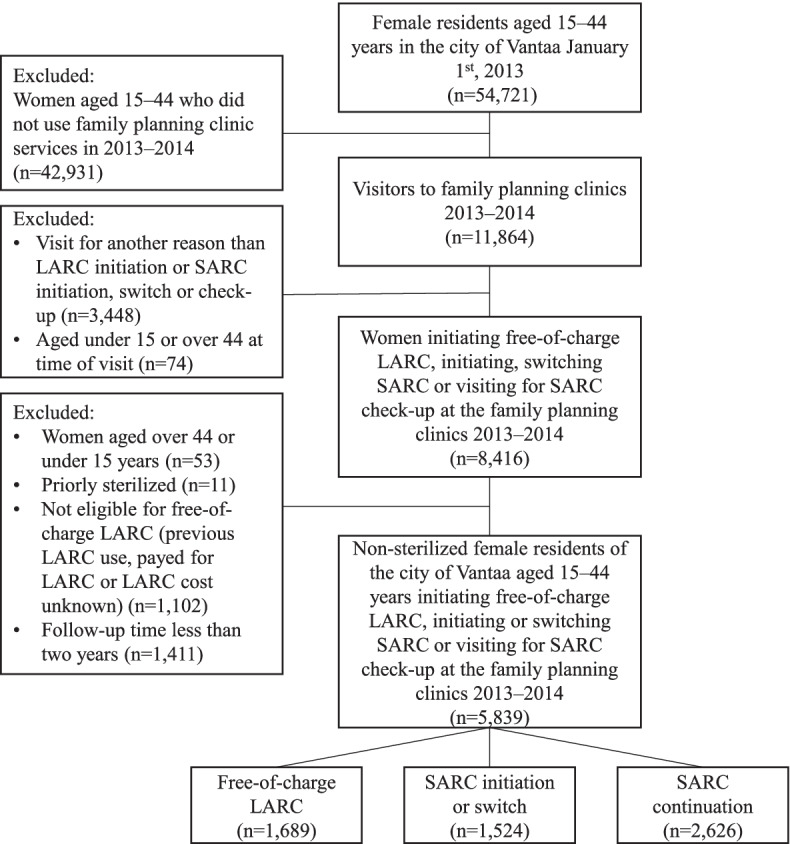
Formation on the study cohorts

This retrospective register-based cohort study included all 15–44-year-old non-sterilized female residents of the city of Vantaa, Helsinki metropolitan area, Finland, who attended the city’s family planning clinics to initiate a LARC method free of charge (*n* = 1689), to initiate or switch to a new SARC method (*n* = 1524) or to continue with their current SARC method (*n* = 2626) in 2013–2014. No data on condom use were available. Since 2013, all women in Vantaa have been offered their first LARC method free of charge at public family planning clinics. Women who had previously used a LARC method were not eligible for a free-of-charge LARC and were thus excluded from the study. Women were followed for 2 years after the index visit regardless of possible discontinuation of the method and were excluded if the follow-up did not extend to 2 years, mainly due to moving away from Vantaa. The formation of study cohorts is illustrated in Fig. 1.

The family planning clinics in Vantaa offer reproductive health services, including contraceptive counselling, screening, and treatment for vaginal and sexually transmitted infections (STIs), counselling on sexuality related questions, abortion referrals, and post-abortion follow-up. Samples for cervical cancer screening are taken only if the woman has not attended PAP smear screening offered by the municipality every 5 years. Public health nurses and midwives provide contraceptive counselling and routine checkups, whilst general practitioners provide consultation, prescriptions, and provision of LARC methods. In addition to LARC methods, women aged under 20 years received a nine-month supply and SARC initiators and switchers a three-month supply of oral contraceptives or contraceptive rings in addition to a prescription for 12 to 18 months.

Women using SARC methods were advised to book a checkup annually or with 18-month intervals, and to contact the clinics if they had problems or wanted to switch method. Women using LARCs were advised to book a checkup within 6 to 12 months (a “thread check”) after initiation in 2013, but since 2014 they were instructed to contact the clinics only in case of concerns, or if they wanted to discontinue or switch method. The checkups included weight and blood pressure measurements, the exclusion of possible emerging contraindications, and discussing satisfaction with the method, but no routine pelvic or breast exams. These checkups were mainly performed by midwifes and public health nurses, and hence did not include diagnostics for medical problems.

We identified women who attended the family planning clinics for the initiation of a free-of charge LARC, the initiation or switch of a SARC method, or SARC checkup from the electronic patient records in Vantaa. We classified these visits using the following recordings: the reason for visit, the procedural codes used to compensate general practitioners, the ATC (Anatomical Therapeutic Chemical classification) drug codes of prescribed contraceptives and the ICPC2-codes (International Classification of Primary Care – 2nd Edition) used to classify reasons for primary care visits. In addition, we (authors TS and FG and a trained study nurse) manually reviewed the LARC visits to obtain the type of LARC chosen, and to verify that the LARC was free of charge.

As the study aimed to evaluate the subsequent use of reproductive health services after initiating free-of-charge LARC, initiating or switching between, or continuing a SARC method, we included characteristics that are associated with both choosing a LARC method and using family planning services (age, history of delivery and induced abortion, being married, socioeconomic status, educational level, having other than the national language as native language/ being non-native, previous gynecological and mental health disorder diagnoses, previous use of family planning services, and prior STIs), according to prior studies [7–14].

To obtain the abovementioned variables, we derived and combined data from the Central Population Register of Vantaa, Statistics Finland, the Finnish Institute of Health and Welfare, and Vantaa’s electronic patient records using a personal identification number assigned to every resident in Finland since the 1960s. The Central Population Register of Vantaa provided data on date of birth, marital status (married/unmarried), and native language (self-reported). Ethnicity or race are not recorded in the national registers, therefore we used native language as a proxy of ethnic variation and defined it as a dichotomous variable of native (Finnish or Swedish) speakers and those speaking other native languages [15]. Statistics Finland provided data on educational attainment and socioeconomic status. Educational attainment was defined according to International Standard Classification of Education and divided into five groups (Table 1). Only educational attainments above basic education are statistically recorded in Finland. Socioeconomic status was divided according to Statistics Finland’s standards into five groups (Table 1).

**Table 1:** Characteristics of women (*n* = 5839) according to their choice of contraception

Characteristic	All participants ***N*** = 5839	LARC initiation ***n*** = 1689	SARC initiation or switch ***n*** = 1524	SARC continuation ***n*** = 2626
**Age, median (IQR)**	24.3 (19.9, 30.5)	28.9 (23.5, 33.9)	20.2 (17.5, 25.2)	23.9 (20.4, 29.3)
**Age categories, years, n** (%)				
15–19	1516 (26.0)	195 (11.5)	744 (48.8)	577 (22.0)
20–24	1641 (28.1)	342 (20.2)	385 (25.3)	914 (34.8)
25–29	1128 (19.3)	390 (23.1)	191 (12.5)	547 (20.8)
30–34	872 (14.9)	428 (25.3)	106 (7.0)	338 (12.9)
35–44	682 (11.7)	334 (19.8)	98 (6.4)	250 (9.5)
**Married, n (%)**	1342 (23.0)	696 (41.2)	183 (12.0)	463 (17.6)
**History of delivery, n** (%)	1801 (30.8)	1134 (67.1)	256 (16.8)	411 (15.7)
**History of induced abortion, n** (%)	876 (15.0)	405 (24.0)	174 (11.4)	297 (11.3)
**History of pregnancy, n (%)**	2246 (38.5)	1267 (75.0)	354 (23.2)	625 (23.8)
**Native language other than Finnish or Swedish, n (%)**	673 (11.5)	304 (18.0)	190 (12.5)	179 (6.8)
**Socioeconomic status**^a^**, n** (%)				
Upper-level employees ^b^	449 (7.7)	191 (11.3)	76 (5.0)	182 (6.9)
Lower-level employees or manual workers ^c^	3337 (57.2)	964 (57.1)	714 (46.9)	1659 (63.2)
Students	1263 (21.6)	231 (13.7)	551 (36.2)	481 (18.3)
Long-term unemployed	331 (5.7)	115 (6.8)	73 (4.8)	143 (5.4)
Entrepreneurs, pensioners, and others not elsewhere classified	443 (7.6)	182 (10.8)	105 (6.9)	156 (5.9)
Unknown	16 (0.3)	6 (0.4)	5 (0.3)	5 (0.2)
**Educational attainment, n (%)**				
Doctoral, master, or equivalent level	287 (4.9)	147 (8.7)	37 (2.4)	103 (3.9)
Bachelor or equivalent level	825 (14.1)	314 (18.6)	106 (7.0)	405 (15.4)
Short-cycle tertiary education	93 (1.6)	33 (2.0)	9 (0.6)	51 (1.9)
Upper secondary education	2582 (44.2)	681 (40.3)	494 (32.4)	1407 (53.6)
Unknown ^d^	2052 (35.1)	514 (30.4)	878 (57.6)	660 (25.1)
**History of STI within the previous year**, n (%)	125 (2.1)	28 (1.7)	41 (2.7)	56 (2.1)
**Visit for gynecological reasons in primary or specialized care or visit in the family planning clinics within the previous year, n (%)**	2925 (50.1)	1157 (68.5)	498 (32.7)	1270 (48.4)
**History of mental health disorder diagnoses in adulthood (ICD-10 codes F10–F16, F18–69, F99)** ^e^**, n (%)**	611 (10.5)	229 (13.6)	160 (10.5)	222 (8.5)
**LARC type, n (%)**				
LNG-IUS		1003 (59.4)	–	–
Implant		533 (31.6)	–	–
Cu-IUD		153 (9.1)	–	–

^a^Socioeconomic status of the youngest age group could also be derived from their family’s socioeconomic status

^b^ Administrative, managerial, professional, and related occupations

^c^ Administrative and clerical occupations or manual workers

^d^ Comprises women with only basic education, as well as without education in Finland, and those not graduating elementary school

^e^ Diagnosed within the previous years in primary or specialized care

*LARC* long-acting reversible contraceptive (i.e. intrauterine device or system or contraceptive implant); *SARC* short-acting reversible contraceptive (i.e. pills, patches or rings); *LNG-IUS* levonorgestrel-releasing intrauterine system;Cu-IUD, copper intrauterine device; *STI* sexually transmitted infections, chlamydia, gonorrhea, or syphilis; *SD* standard deviation; *ICD-10* The International Statistical Classification of Diseases and Related Health Problems, 10th Revision

The groups differed significantly for all variables except for history of STI tested with T-test for continuous variables, χ^2^-test for categorical variables

From the Finnish Institute of Health and Welfare we obtained data on births, induced abortions, sterilizations, STIs, outpatient visits, and hospital-care episodes and care episodes in primary health care both before and during follow-up. These registers are comprehensive, validated, and of high quality [16–18]. We included all births and induced abortions available in the registers since 1987 and the diagnoses of STIs within the previous year and during follow-up from the register of infectious diseases with mandatory reporting of all laboratory diagnosed STIs. The International Statistical Classification of Diseases and Related Health Problems, 10th Revision (ICD-10) diagnoses are registered at visits in primary and specialized care, and on hospital care episodes, and additionally ICPC2-codes are used at primary care visits. We obtained the diagnoses of mental health disorders in adulthood (ICD-10 codes F10–69 and F99, excluding nicotine dependence, F17) and gynecological morbidities, including both ICD-10 diagnostic codes and ICPC-2 codes recorded at these episodes.

We derived data on previous visits in the family planning clinics from the patient records in Vantaa. To adjust for the previous need of reproductive health services, we combined the history of gynecological diagnoses in primary or specialized care, visiting the family planning clinics and the diagnoses of STIs all within the previous year as a combined variable analyzed dichotomously — history or no history.

We obtained data on visits during the 2 years of follow-up at the family planning clinics from the patient records. We classified these visits according to the reason for visit, ATC drug codes and ICPC2-codes into visits for routine checkup or for other reasons such as concerns with the method or side effects, or for abortion care, or LARC procedures (See Supplementary Table 1, Additional file 1). Additionally, we included data from the registers of The Finnish Institute of Health and Welfare on visits for gynecological reasons in primary and specialized care within the two-year follow-up. We classified these visits as shown in Supplementary Table 1 according to ICD-10, ICPC2, and the medical and surgical procedures according to Nordic Medico-Statistical Committee (NOMESCO) Classification of Surgical Procedures at all visits. For each woman, we calculated the overall number of visits that occurred within 2 years for gynecological reasons in specialized or primary care following the index date. In addition, to evaluate the additional use of reproductive health services beyond routine checkups, we retrieved the number of visits for reasons other than routine checkups at the family planning clinics.

We described characteristics of the study population divided into three groups: free-of-charge LARC initiators, SARC initiators or switchers, and SARC continuers. We analyzed the number of visits for various reasons as counts and percentages, and calculated incidence rate per 100 person years with 95% confidence intervals (CIs) for each reason.

We fitted negative binomial regression models on visit counts to assess differences in service use between the three groups. As the visit counts demonstrated overdispersion, we chose to model the data using the negative binomial regression instead of the commonly used Poisson regression for counts. We performed model evaluation and found the negative binomial regression model to fit the data well. To control for confounding, we included all the above-mentioned variables previously identified to associate both with choosing a LARC and using family planning services in the initial model. We examined the dependencies between these confounding variables, the outcome, and the variable of interest by drawing a DAG (directed acyclic graph) (Supplementary Figure 1, Additional file). As seen in the DAG, the dependencies between the factors explored as confounders were complex. We aimed to simplify the multivariate model to avoid these background dependencies from obscuring the results and to avoid overfitting. Thus, we chose to use stepwise selection to produce a simple, yet representative model. First, with backward selection we removed variables from the full model that had less than 10% effect on the estimate. We then continued by adding the removed variables one-by-one back to the model. If the variable changed the estimates by 10% or more, it was kept in the model. This process resulted in a model including categorical age, the history of pregnancy (history of delivery and induced abortion combined), and the use of reproductive services within the previous year (visits at the family planning clinics, and in primary or specialized care for gynecological reasons and having had an STI within the previous year combined). To assess the stability of the reported model, we repeated the model selection process for ten randomly generated datasets. To preserve statistical power, we generated ten random datasets of equal the size to the original data by using random sampling with replacement. For eight out of ten datasets, the model selection yielded the same model (our final model). Once prior pregnancies were left out, and once marital status was included in the model, but all other variables remained the same. Thus, we considered the model selection process to be reasonably stable.

We reported crude and adjusted incidence rate ratios (IRRs) for service use with 95% CIs from the final model. We checked for multicollinearity in the models by calculating variance inflation factors (VIFs) both for the primary, full, multivariate model and for the final negative binomial regression model. VIFs were low, under 3.5, for all variables, thus showing no problematic multicollinearity in either model.

To evaluate how age modifies service use, we analyzed visits for all gynecological reasons in primary or specialized care, and visits for other reasons than routine checkups at the family planning clinics in different age groups. We calculated counts and percentages, and incidences per 100 woman-years with 95% CIs, and adjusted IRRs with the same binomial regression model as for the whole cohort.

We also analyzed subsequent visits grouped by different LARC methods as counts and percentages, calculated incidences per 100 woman-years with 95% CIs and calculated adjusted IRRs with the same binomial regression model as for the whole cohort.

We set the significance level at 0.05 (5%). All analyses were conducted using statistical software R (version 4.0.2) [19].

## Results

We followed altogether 5839 non-sterilized women aged 15–44 years. Of those 29% initiated free-of-charge LARC methods (*n* = 1689), 26% initiated or switched SARC methods (*n* = 1524), and 45% continued with the same SARC method (*n* = 2626) at the index visit. Compared to SARC initiators, switchers or continuers, free-of-charge LARC initiators were older, and more often parous or had a history of induced abortion. They were also more often upper-level employees, or of the highest educational level, and had attended health care more frequently within the previous year for gynecological reasons or mental health disorders (Table 1).

Of the 5839 women followed for 2 years, 4397 (75.3%) had at least one additional visit. A total of 11,290 visits accumulated, of which 7260 (64.3%) were visits at family planning clinics, 3385 (30.0%) visits for gynecological reasons in primary care, and 645 (5.7%) visits for gynecological reasons in specialized health care. Of the family planning clinic visits 3804 (52.4%) were for routine checkup, and 3456 (47.6%) for other reasons such as abnormal uterine bleeding, method switching, or discontinuation. During follow-up, there were altogether 96.1 visits (95% CI 92.8–99.5) per 100 woman-years among free-of-charge LARC initiators, 124.5 visits (95% CI 120.6–128.5) per 100 woman-years among SARC initiators or switchers, and 80.9 visits (95% CI 78.5–83.4) per 100 woman-years among SARC continuers (Table 2).

**Table 2:** Visits in primary and specialized care and at the family planning clinics for gynecological reasons

	Visited n (%)	Total number of visits	Visits per 100 woman-years	Crude IRR (95%CI)	Adjusted IRR (95%CI)
**All visits for gynecological reasons**^a^ **and all visits in family planning clinics**					
LARC initiation	1160 (68.7)	3247	96.1 (92.8–99.5)	0.77 (0.72–83)	0.86 (0.79–0.93)
SARC initiation or switch	1245 (81.7)	3795	124.5 (120.6–128.5)	Ref.	Ref.
SARC continuation	1992 (75.9)	4248	80.9 (78.5–83.4)	0.65 (0.61–0.69)	0.72 (0.67–0.77)
All	4397 (75.3)	11,290	96.7 (94.9–98.5)	Not applicable	Not applicable
**All visits for gynecological reasons**^a^ **and other than routine checkups at family planning clinics**					
LARC initiation	839 (49.7)	2545	75.3 (72.4–78.3)	0.96 (0.87–1.07)	0.93 (0.82–1.05)
SARC initiation or switch	892 (58.5)	2559	78.1 (75.0–81.4)	Ref.	Ref.
SARC continuation	1094 (41.7)	2382	48.7 (46.9–50.6)	0.62 (0.56–0.69)	0.65 (0.59–0.72)
All	2825 (48.4)	7486	64.1 (62.7–65.6)	Not applicable	Not applicable
**Routine checkups at family planning clinics**					
LARC initiation	660 (39.1)	702	20.8 (19.3–22.4)	0.45 (0.41–0.49)	0.65 (0.58–0.72)
SARC initiation or switch	867 (56.9)	1413	46.4 (44.0–48.8)	Ref.	Ref.
SARC continuation	1446 (55.1)	1689	32.2 (30.6–33.7)	0.69 (0.65–0.74)	0.84 (0.77–0.90)
All	2973 (50.9)	3804	32.6 (31.5–33.6)	Not applicable	Not applicable
**Other than routine checkup at family planning clinics**					
LARC initiation	631 (37.4)	1199	35.5 (33.5–37.6)	0.94 (0.84–1.06)	1.03 (0.90–1.18)
SARC initiation or switch	645 (42.3)	1146	37.6 (35.5–39.8)	Ref.	Ref.
SARC continuation	707 (26.9)	1111	21.2 (19.9–22.4)	0.56 (0.50–0.63)	0.61 (0.55–0.69)
All	1983 (34.0)	3456	29.6 (28.6–30.6)	Not applicable	Not applicable

*LARC* long-acting reversible contraception; *SARC* short-acting reversible contraception; *CI* confidence interval; *IRR* incidence rate ratio, calculated with negative binomial regression, adjusted with categorical age, history of pregnancy and history of sexually transmitted infection or visit for gynecological reasons in primary or specialized health care or visit at the family planning clinics within the previous year

^a^In primary or specialized health care

After excluding the routine checkup visits, free-of-charge LARC initiators had similar adjusted IRR 0.93 (95% CI 0.82–1.05) for additional visits for gynecological reasons in primary or specialized care, or for other reasons at the family planning clinics compared with SARC initiators or switchers. In contrast, SARC continuers had lower adjusted IRRs 0.65 (95% CI 0.59–0.72) for these additional visits compared with SARC initiators or switchers (Table 2).

Both SARC groups had more routine checkup visits compared with free-of-charge LARC initiators (56.9 and 55.1% vs 39.1%) (Table 2). There was no difference in the rate of checkup visits between years 2013 and 2014 in the LARC group (40.0% vs 38.5%) even though women were advised to attend a checkup in 2013 but not in 2014. Among SARC initiators or switchers, 7.5% and among SARC continuers 5.0% had a LARC procedure, namely LARC insertion, at the family planning clinics during the two-year follow-up.

**Fig. 2:**
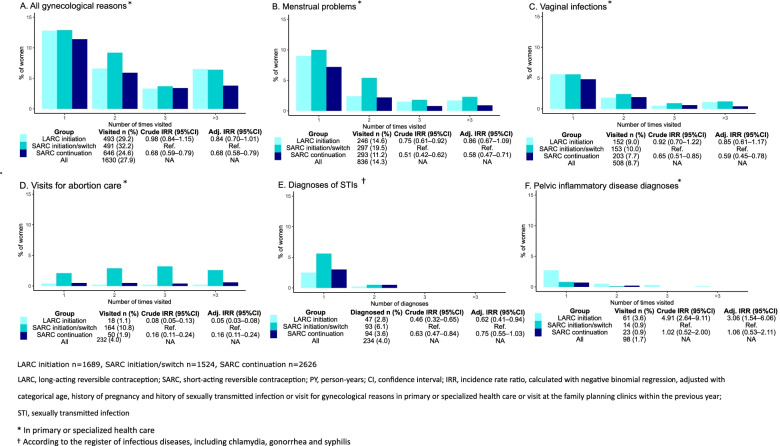
Use of reproductive health services in primary and specialized care for gynecological reasons by study groups

As shown in Table 3 and Fig. 2, free-of-charge LARC initiators used health services for all gynecological reasons similarly as SARC initiators or switchers (adjusted IRR 0.84, 95% CI 0.70–1.01). This included menstrual problems (IRR 0.86, 95% CI 0.69–1.09), and vaginal infections (IRR 0.85, 95% CI 0.61–1.17). However, compared with SARC initiators or switchers, SARC continuers used the services less for all gynecological reasons (IRR 0.68, 95 CI 0.58–0.79), especially menstrual problems (IRR 0.58, 95% CI 0.47–0.71) and vaginal infections (IRR 0.59, 95% CI 0.45–0.78).

**Table 3:** Visits for various gynecological reasons in primary and specialized care

Reason for visiting	Total number of visits	Visits per 100 woman-years (95% CI)
**All gynecological reasons**^a^		
LARC initiation	1346	39.8 (37.7–42.0)
SARC initiation or switch	1236	40.6 (38.3–42.9)
SARC continuation	1448	27.6 (26.2–29.0)
All	4030	34.5 (33.5–35.6)
**Menstrual problems**^a^		
LARC initiation	465	13.8 (12.5–15.1)
SARC initiation or switch	562	18.4 (16.9–20.0)
SARC continuation	493	9.4 (8.6–10.3)
All	1520	13.0 (12.4–13.7)
**Vaginal infections (such as candida or bacterial vaginosis)**^a^		
LARC initiation	294	8.7 (7.7–9.8)
SARC initiation or switch	288	9.4 (8.4–10.6)
SARC continuation	325	6.2 (5.5–6.9)
All	907	7.8 (7.3–8.3)
**Visits for abortion care at family planning clinics or specialized health care**		
LARC initiation	42	1.2 (0.9–1.7)
SARC initiation or switch	476	15.6 (14.2–17.1)
SARC continuation	135	2.6 (2.2–3.0)
All	653	5.6 (5.2–6.0)
**Diagnoses of STIs**^b^		
LARC initiation	51	1.5 (1.1–2.0)
SARC initiation or switch	101	3.3 (2.7–4.0)
SARC continuation	109	2.1 (1.7–2.5)
All	261	2.2 (2.0–2.5)
**Pelvic inflammatory disease diagnoses**^a^		
LARC initiation	87	2.6 (2.1–3.2)
SARC initiation or switch	16	0.5 (0.3–0.9)
SARC continuation	28	0.5 (0.4–0.8)
All	131	1.1 (0.9–1.3)

*LARC* long-acting reversible contraception; *SARC* short-acting reversible contraception; *CI* confidence interval; *STI* sexually transmitted infection

^a^In primary or specialized health care

^b^According to the register of infectious diseases, including chlamydia, gonorrhea, and syphilis

Free-of-charge LARC initiators and SARC continuers used services less for abortion care (adjusted IRRs 0.05, 95% CI 0.03–0.08 and 0.16, 95% CI 0.11–0.24, respectively) than SARC initiators or switchers. On the other hand, free-of-charge LARC initiators had more visits with diagnosis of pelvic inflammatory disease (PID) (adjusted IRR 3.06, 95% CI 1.54–6.06), compared with SARC initiators or switchers. Free-of-charge LARC initiators were less often diagnosed with STIs during the two-year follow up (adjusted IRR 0.62, 95% CI 0.41–0.94) than SARC initiators or switchers (Fig. 2).

In analyses according to different age groups (See Supplementary Table 2 and Supplementary Fig. 2, Additional file 1), the youngest women used the services most for all gynecological reasons and other reasons than routine checkups. The service use decreased with increasing age. In absolute numbers, under 30 year-old LARC initiators used services more than same-aged SARC initiators or switchers. This difference subsided after adjustment for prior pregnancies and service use within the previous year. We found no difference in service use between women aged 30–34 years initiating LARC methods or initiating, switching, or continuing SARC methods. In all other age groups, SARC continuers used services less than LARC initiators, or SARC initiators or switchers.

**Fig. 3:**
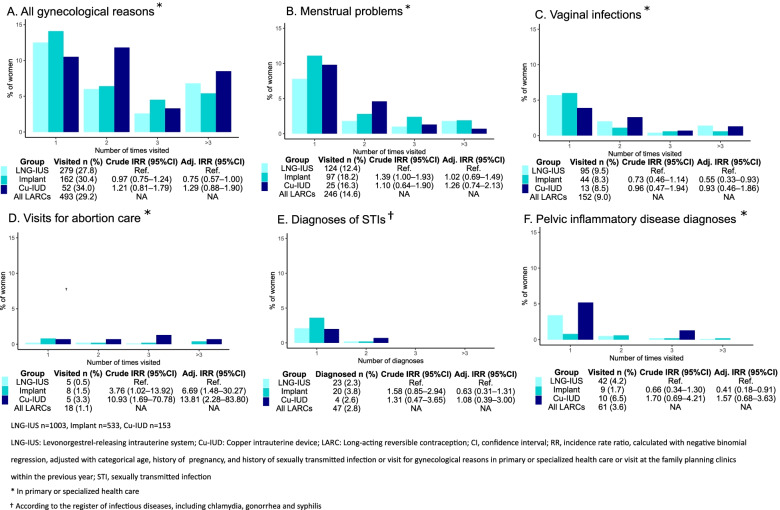
Use of reproductive health services for various gynecological reasons according to choice of free-of-charge long-acting reversible contraceptive (LARC) method

In the analyses concerning differences in characteristics and service use among women initiating different LARC methods (See Supplementary Tables 3, 4 and 5, and Fig. 3, Additional file 1), women choosing implants were younger, more often nulliparous, unmarried, and more often had visits for gynecological reasons in primary or specialized care or at the family planning clinics or experienced more STIs in the previous year than IUD users. Women using Cu-IUD had more visits for other reasons than routine checkups at the family planning clinics (adjusted IRR 1.48 [95% CI 1.12–1.94]), but no difference in visits for gynecological reasons in primary or specialized care compared to the users of LNG-IUS. Women choosing Cu-IUDs needed abortion care more often than women choosing LNG-IUS (adjusted IRR 13.81, 95% CI 2.28–83.80). Women choosing contraceptive implants had fewer visits for routine checkups (adjusted IRR 0.41, 95% CI 0.33–0.52), for vaginal infections (adjusted IRR 0.55, 95% CI 0.33–0.93) or PID diagnoses (adjusted IRR 0.41, 95% CI 0.18–0.91), but more visits for abortion care (adjusted IRR 6.69, 95% CI 1.48–30.24) than women choosing LNG-IUS.

## Discussion

We found that free-of-charge LARC initiators attended reproductive health services for method-related or gynecological reasons similarly as SARC initiators or switchers. In contrast, SARC continuers attended these services less.

Women using SARC methods attended more routine checkups than those initiating LARC methods. Still, even if advised only 55% of SARC users attended a checkup within 2 years. This might imply a low rate of continuation as prescriptions were valid for 1 year. Nevertheless, 80% of women using SARCs used reproductive health services during follow-up, and the prescriptions could have been renewed at any of these visits. Among LARC users 69% used services during follow-up. A previous study on these same LARC initiators showed high method continuation rates, over 70% at 2 years [20].

Free-of-charge LARC initiators had less need for abortion care. This is consistent with previous studies showing a significantly lower need of induced abortion among women choosing free-of-charge LARC over other contraceptive options, and the superior contraceptive efficacy of LARC methods compared to SARC methods [1, 5, 21]. Interestingly, SARC continuers had lower need for abortion care than SARC initiators or switchers, and only slightly higher need than free-of-charge LARC initiators. As continuing users are likely to be satisfied with their method, this finding underlines the importance of satisfaction in adherence and thus with the contraceptive effectiveness of short-acting methods. While the rate of abortion declined most among women under 25 years of age after the free-of-charge LARC program was implemented [22], our study shows that women under 30 initiating LARC methods used reproductive health services more than same aged women initiating SARC methods. Young women may be more inclined to react to common side effects of LARC methods, and hence need more services.

Reassuringly, the rates of STIs were similar among free-of-charge LARC initiators and SARC users, and among women initiating different LARC methods. Previous studies suggest that LARC users may be less likely to use condoms as dual protection compared to women using less effective contraceptive options such as SARCs making them to be at higher risk to contract sexually transmitted infections [23–27]. However, results in previous studies comparing the occurrence of STIs is between LARC users and SARC users are conflicting [23, 27, 28]. The CHOICE study found increased odds of STIs among LARC initiators, whereas a study on adolescents in New York reported a similar rate of chlamydia infections among LARC users and non-users [27, 28]. Both these studies offered annual testing for STIs. In our data, STI testing was mainly based on women requesting it or having STI-related symptoms. The women using SARC methods in our study were possibly more frequently offered STI testing, as they attended more checkups.

Free-of-charge LARC initiators had more visits with the diagnosis of PID than SARC users, and women choosing IUDs had more of these visits than women choosing contraceptive implants. Clinical diagnosis of PID is a difficult one and clinicians may be prone to suspecting PIDs among IUD users as pelvic pain is a common adverse event of IUDs [20, 29, 30]. Thus distinguishing IUD-related pelvic pain clinically from true PID can be challenging [31].

We found that women choosing using Cu-IUD had more visits for other reasons than routine checkups compared to women using LNG-IUS. This is in line with a previous study comparing LARC methods, where Cu-IUD users had higher rate of discontinuation due to increased bleeding and cramping [20]. This is likely to explain why women using Cu-IUD also had a higher need for abortion care. On the other hand, the superior contraceptive efficacy of the LNG-IUS compared to the Cu-IUD is in line with the prospective EURAS-IUD study [32].

The study has several strengths. Previous research on free-of-charge LARC programs has focused on effects on abortion rates and discontinuation, but not on the overall use of reproductive health services, which we were able to assess with real-world data from high quality national registers. The study groups differed significantly., as the users of LARC and SARC methods do in real-life settings. However, we were able to control for confounding with several factors through adjustment. We identified confounders, carefully chose the regression model used and tested that it fit the data.

Our study has also limitations. Residual confounding due to unmeasured or unknown factors cannot be ruled out. We did not have data on ethnicity or immigrant status but used native language as a proxy for these characteristics. Women born in Finland with no educational attainments in the registers have completed a mandatory nine-year basic education. However, for immigrant women, the unknown educational attainment represents a true unknown level if they have migrated to Finland after the age of 16 when the individual is no longer legally required to attend school.

Moreover, we had no data on the pregnancy intentions of the women. Pregnancy intentions influence contraceptive choice and adherence with the method as women with positive predisposition towards pregnancy tend to choose less reliable contraceptive methods and use methods less consistently [33–35]. Additionally, women switching method are likely to have had problems with their previous method, and thus be more likely to have additional need for services. Equally, women initiating methods such as LNG-IUS for menstrual problems are likely to have further service needs. Although, the data represents service use in Vantaa well, in our population cost was a minimal barrier for service use, and the family planning services offed by Vantaa comprehensive. This may not be the case in other healthcare settings and affects the generalizability of our results. Moreover, we were unable to include possible appointments in the private sector, as these visits are not recorded in the registers of the Finnish Institute of Health and Welfare [36]. In 2013, 18% of women of all ages in the city of Vantaa attended a private obstetrician-gynecologist [37]. Thera are no data on which proportion of these appointments regarded contraception or were by women included in our study. The LARC users were older and of a higher socioeconomic status, and thus likely to have had better access to high-cost private services. Therefore, we may have underestimated the service use more among LARC users than among SARC users. In this study, we focused on the service use of first-time LARC users who had the possibility to initiate the method free-of-charge. Further research comparing the use of reproductive health services between women continuing SARC methods and LARC methods is needed.

## Conclusions

While free-of-charge LARC initiators have lower need for abortion care compared to SARC initiators or switchers, women initiating both LARC and SARC methods have similar overall need for reproductive health services. Women continuing with their SARC method need abortion care and overall reproductive health services less than women initiating a new SARC method. This information is pivotal for implementation and design of free-of-charge LARC programs and in ensuring adequate services for women initiating LARC and SARC methods both to support continuation by counseling with method-related issues, and to assure reproductive autonomy.

## Supplementary Information

**Additional file 1.**

## Data Availability

We cannot share the data according to the ethical approval and permissions given by the register-keeping authorities. Similar data, except the patient records in Vantaa, can be applied from Findata, the Health and Social Data Permit Authority in Finland: www.findata.fi/en/

## Abbreviations

LARC: Long-acting reversible contraceptive (i.e. intrauterine device or system, or contraceptive implant)
SARC: Short-acting reversible contraceptive (i.e. pills, patches or rings)
LNG-IUS: Levonorgestrel-releasing intrauterine system
Cu-IUD: Copper intrauterine device
STI: Sexually transmitted infections, chlamydia, gonorrhea, or syphilis
ATC: Anatomical Therapeutic Chemical classification drug codes of prescribed contraceptives
ICPC2-codes: International Classification of Primary Care – 2nd Edition ICD-10, The International Statistical Classification of Diseases and Related Health Problems, 10th Revision
CI: Confidence interval
IRR: Incidence rate ratio
PID: Pelvic inflammatory disease
