# Supplementary material for: Use of reproductive health services among women using long- or short-acting contraceptive methods – a register-based cohort study from Finland
Source: BMC Public Health. 2022 Jun 14;22:1185. doi: 10.1186/s12889-022-13581-3 (PMC9199191; doi:10.1186/s12889-022-13581-3)
Supplement: Supplementary file 1 — Additional file 1. [file 12889_2022_13581_MOESM1_ESM.docx]

**Additional files:**

Additional file 1: Additional file_1.doc, Supplementary Tables 1-5 and Supplementary Figures 1 & 2

**Supplementary Table 1. ICD-10, ICPC2 and NCSP codes used to identify the reasons for follow-up visits.**

|  | **ICD-10, ICPC2 and NCSP codes** |
| --- | --- |
| **All visits for gynecological reasons in primary or specialized care and all visits in family planning clinics** | ICD-10: N7, N8, N90, N91, N92, N93, N94, N95, N96, N97, N98, Z30, Z31  ICPC2: W15, X01, X02, X03, X04, X05, X06, X07, X08, X09, X13, X14, X15, X16, X17, X24, X27, X28, X29, X72, X74  All visits at the family planning clinics from the family planning clinics registers |
| **All visits for gynecological reasons in primary or specialized care and other than routine checkup visits at family planning clinics** | Same diagnostic codes as above, but only visits defined as non-follow-up from the family planning clinics records. |
| **Routine checkup visits at the family planning clinics** | All visits in family planning clinics identified by the registered reason for visit as follow-up |
| **Visits at the family planning clinics for other reasons than routine checkup** | All visits in family planning clinics identified by the registered reason for visit as non-follow-up |
| **Visits for gynecological reasons in primary or specialized care** | ICD-10: N7, N8, N90, N91, N92, N93, N94, N95, N96, N97, N98, Z30, Z31  ICPC2: W15, X01, X02, X03, X04, X05, X06, X07, X08, X09, X13, X14, X15, X16, X17, X24, X27, X28, X29, X72, X74 |
| **Visits for menstrual problems in primary or specialized care** | ICD-10: N92, N93, N94,  ICPC2: X02, X03, X04, X05, X06, X07, X08,  X09, X10, X13, X14 |
| **Visits for vaginal infections in primary or specialized care** | ICD-10: N76.0, N76.1, N76.2, N76.3, N76.8  ICPC2: X15, X72, X84 |
| **Visits for abortion care in specialized care or at family planning clinics** | ICD-10: O04, O05, O07  ICPC2: W83 |
| **Diagnoses of STIs** | Based on register of infectious diseases maintained by the Finnish Institute of Health and Welfare |
| **Visits for pelvic inflammatory diagnoses in primary or specialized care** | ICD-10: N70, N71, N72, N73  ICPC2: X74 |
|  |  |

ICD-10, The International Statistical Classification of Diseases and Related Health Problems, 10^th^ Revision

ICPC2, International Classification of Primary Care – 2nd Edition

NCSP = Nordic Medico-Statistical Committee (NOMESCO) Classification of Surgical Procedures Classification of Surgical Procedures

LARC, long-acting reversible contraception

STI, sexually transmitted infections, chlamydia, gonorrhea, or syphilis

**Supplementary Figure 1. A DAG (directed acyclic graph) illustrating the dependencies between the confounding variables, the outcome (subsequent use of reproductive health services) and the variable of interest (selecting the method of contraception).**

**
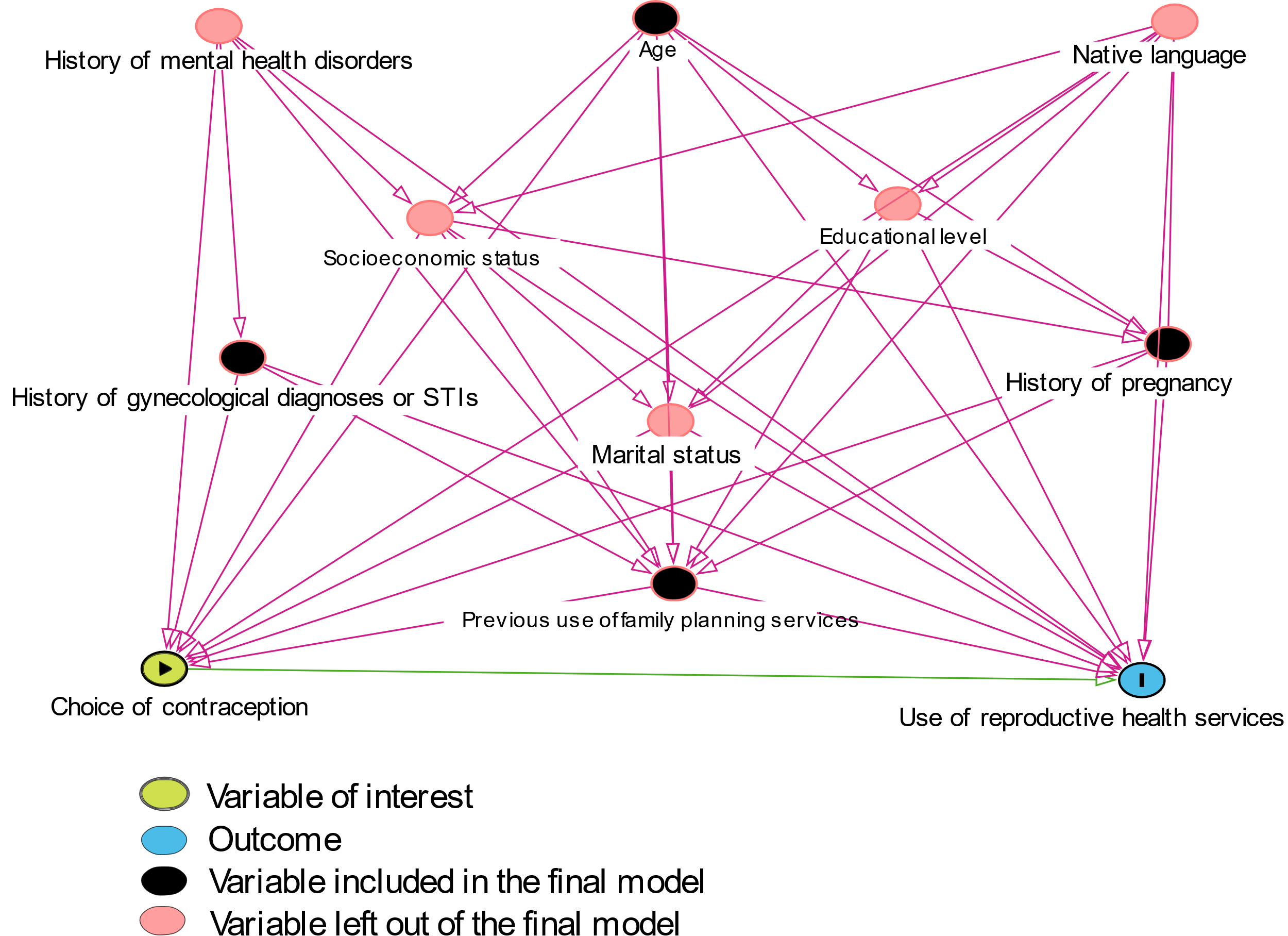
**

**Supplementary Table 2. Visits in five age groups for gynecological reasons in primary and specialized care according to study groups.**

| **Age group** | **Number of women** | **Visited**  **n (%)** | **Total number of visits** | **Visits per 100 woman-years (95% CI)** | **Crude IRR (95%CI)** | **Adjusted IRR (95%CI)** |
| --- | --- | --- | --- | --- | --- | --- |
| **15–19 years** |  |  |  |  |  |  |
| LARC initiation | 195 | 128 (65.6) | 430 | 110.3 (100.1–121.2) | 1.23 (1.01–1.51) | 0.88 (0.70–1.09) |
| SARC initiation or switch | 744 | 485 (65.2) | 1,330 | 89.4 (84.6–94.3) | Ref. | Ref. |
| SARC continuation | 577 | 300 (52.0) | 723 | 62.7 (58.2–67.4) | 0.70 (0.60–0.81) | 0.58 (0.50–0.68) |
| All | 1,516 | 913 (60.2) | 2,843 | 93.8 (90.4–97.3) | Not applicable | Not applicable |
| **20­–24 years** |  |  |  |  |  |  |
| LARC initiation | 342 | 208 (60.8) | 683 | 99.9 (92.5–107.6) | 1.25 (1.01–1.56) | 1.05 (0.83–1.32) |
| SARC initiation or switch | 385 | 216 (56.1) | 613 | 79.6 (73.4–86.2) | Ref. | Ref. |
| SARC continuation | 914 | 383 (41.9) | 910 | 49.8 (46.6–53.1) | 0.63 (0.52–0.75) | 0.65 (0.54–0.78) |
| All | 1,641 | 807 (49.2) | 2,206 | 67.2 (64.4–70.1) | Not applicable | Not applicable |
| **25–29 years** |  |  |  |  |  |  |
| LARC initiation | 390 | 207 (53.1) | 598 | 76.7 (70.6–83.1) | 1.30 (0.98–1.72) | 1.06 (0.79–1.43) |
| SARC initiation or switch | 191 | 94 (49.2) | 225 | 58.9 (51.5–67.1) | Ref. | Ref. |
| SARC continuation | 547 | 207 (37.8) | 452 | 41.3 (37.6–45.3) | 0.70 (0.53–0.92) | 0.70 (0.53–0.93) |
| All | 1,128 | 509 (45.0) | 1,275 | 56.5 (53.5–59.7) | Not applicable | Not applicable |
| **30–34 years** |  |  |  |  |  |  |
| LARC initiation | 428 | 170 (39.7) | 461 | 53.9 (49.1–59.0) | 1.07 (0.72–1.58) | 0.86 (0.57–1.28) |
| SARC initiation or switch | 106 | 48 (45.3) | 107 | 50.5 (41.4–61.0) | Ref. | Ref. |
| SARC continuation | 338 | 128 (37.9) | 317 | 46.9 (41.9–52.4) | 0.93 (0.62–1.40) | 0.90 (0.60–1.36) |
| All | 872 | 346 (39.7) | 885 | 50.7 (47.5–54.2) | Not applicable | Not applicable |
| **35–44 years** |  |  |  |  |  |  |
| LARC initiation | 334 | 128 (37.7) | 373 | 55.8 (50.3–61.8) | 1.02 (0.67–1.56) | 0.87 (0.56–1.35) |
| SARC initiation or switch | 98 | 49 (50.0) | 107 | 54.6 (44.7–66.0) | Ref. | Ref. |
| SARC continuation | 250 | 76 (30.4) | 157 | 31.4 (26.7–36.7) | 0.58 (0.37–0.90) | 0.57 (0.36–0.90) |
| All | 682 | 251 (36.8) | 637 | 46.7 (43.1–50.5) | Not applicable | Not applicable |

LARC, long-acting reversible contraception

SARC, short-acting reversible contraception

CI, confidence interval

IRR, incidence rate ratio, calculated with negative binomial regression, adjusted with history of pregnancy and history of sexually transmitted infection, or visit for gynecological reasons in primary or specialized health care or visit at the family planning clinics within the previous year

**Supplementary Figure 2. Visit rate with 95% confidence intervals (CI) in primary and specialized care for all gynecological reasons and for other reasons than routine checkups at family planning clinics according to five age groups and study groups.**

**
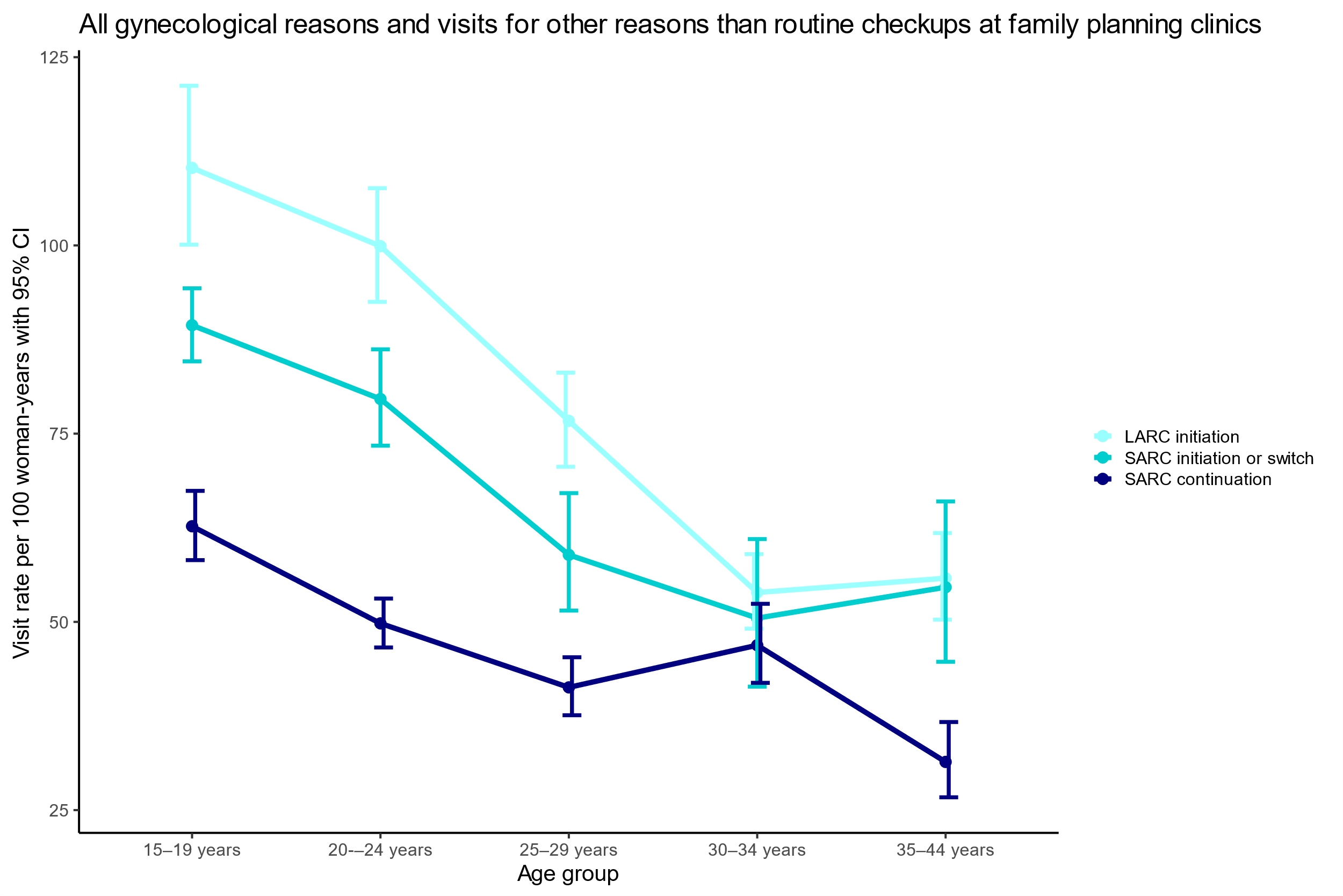
**

**Supplementary Table 3. Characteristics of women according to the type of LARC method initiated.**

| **Characteristic** | **LNG-IUS**  **(n=** **1,003)** | **Implant**  **(n=** **533)** | **Cu-IUD**  **(n=** **153)** |
| --- | --- | --- | --- |
| **Age, median (IQR)** | 31.2 (26.7, 35.5) | 23.2 (19.3, 29.7) | 28.3 (25.0, 32.8) |
| **Age categories, years**, **n (%)** |  |  |  |
| 15–19 | 34 (3.4) | 157 (29.5) | 4 (2.6) |
| 20–24 | 150 (15.0) | 157 (29.5) | 35 (22.9) |
| 25–29 | 245 (24.4) | 93 (17.4) | 52 (34.0) |
| 30–34 | 300 (29.9) | 83 (15.6) | 45 (29.4) |
| 35–44 | 274 (27.3) | 43 (8.1) | 17 (11.1) |
| **Married**, **n (%)** | 494 (49.3) | 127 (23.8) | 75 (49.0) |
| **History of delivery**, **n (%)** | 832 (83.0) | 190 (35.6) | 112 (73.2) |
| **History of induced abortions**, **n (%)** | 269 (26.8) | 96 (18.0) | 40 (26.1) |
| **History of pregnancy, n (%)** | 898 (89.5) | 245 (46.0) | 124 (81.0) |
| **Native language other than Finnish or Swedish, n (%)** | 162 (16.2) | 87 (16.3) | 55 (35.9) |
| **Socioeconomic status***, **n (%)** |  |  |  |
| Upper-level employees ^†^ | 141 (14.1) | 29 (5.4) | 21 (13.7) |
| Lower-level employees or manual workers ^‡^ | 602 (60.0) | 277 (52.0) | 85 (55.6) |
| Students | 80 (8.0) | 132 (24.8) | 19 (12.4) |
| Long-term unemployed | 69 (6.9) | 34 (6.4) | 12 (7.8) |
| Entrepreneurs, pensioners, and others not elsewhere classified | 106 (10.6) | 60 (11.3) | 16 (10.5) |
| Unknown | 5 (0.5) | 1 (0.2) | 0 (0.0) |
| **Educational attainment, n (%)** |  |  |  |
| Doctoral, master, or equivalent level | 115 (11.5) | 14 (2.6) | 18 (11.8) |
| Bachelor or equivalent level | 231 (23.0) | 51 (9.6) | 32 (20.9) |
| Short-cycle tertiary education | 29 (2.9) | 3 (0.6) | 1 (0.7) |
| Upper secondary education | 395 (39.4) | 225 (42.2) | 61 (39.9) |
| Unknown ^§^ | 233 (23.2) | 240 (45.0) | 41 (26.8) |
| **History of STI within the previous year, n (%)** | 9 (0.9) | 19 (3.6) | 0 (0.0) |
| **Visit for gynecological reasons in primary or specialized care or visit in the family planning clinics within the previous year, n (%)** | 621 (61.9) | 429 (80.5) | 107 (69.9) |
| **History of mental health disorder diagnoses in adulthood (ICD-10 codes F10–F16, F18-69, F99) ^\|\|^, n (%)** | 120 (12.0) | 90 (16.9) | 19 (12.4) |

LARC, long-acting reversible contraceptive (i.e. intrauterine device or system or contraceptive implant)

LNG-IUS, levonorgestrel-releasing intrauterine system

Cu-IUD, copper intrauterine device

STI, sexually transmitted infections, chlamydia, gonorrhea, or syphilis

IQR, interquartile range

ICD-10, The International Statistical Classification of Diseases and Related Health Problems, 10^th^ Revision

*Socioeconomic status of the youngest age group could also be derived from their family’s socioeconomic status.

^†^ Administrative, managerial, professional, and related occupations

^‡^ Administrative and clerical occupations or manual workers

^§^ Comprises women with only basic education, as well as without education in Finland, and those not graduating elementary school

^||^ Diagnosed within the previous years in primary or specialized care

The groups differed significantly for all variables tested with T-test for continuous variables, χ2-test for categorical variables

**Supplementary Table 4. Visits in primary and specialized care for gynecological reasons and visits at the family planning clinics according to choice of LARC method.**

|  | **Visited**  **n (%)** | **Total number of visits** | **Visits per 100 woman-years (95%CI)** | **Crude IRR (95%CI)** | **Adjusted IRR (95%CI)** |
| --- | --- | --- | --- | --- | --- |
| **All visits for gynecological reasons* and all visits in family planning clinics** |  |  |  |  |  |
| LNG-IUS | 705 (70.3) | 1,893 | 94.4 (90.2–98.7) | Ref. | Ref. |
| Implant | 332 (62.3) | 980 | 91.9 (86.3–97.9) | 0.97 (0.86–1.11) | 0.77 (0.66–0.89) |
| Cu-IUD | 123 (80.4) | 374 | 122.2 (110.1–135.3) | 1.30 (1.06–1.59) | 1.23 (1.01–1.50) |
| All LARCs | 1,160 (68.7) | 3,247 | 96.1 (92.8–99.5) | Not applicable | Not applicable |
| **All visits for gynecological reasons* and other than routine checkups at family planning clinics** |  |  |  |  |  |
| LNG-IUS | 460 (45.9) | 1,399 | 69.7 (66.1–73.5) | Ref. | Ref. |
| Implant | 290 (54.4) | 851 | 79.8 (74.6–85.4) | 1.14 (0.96–1.36) | 0.90 (0.74–1.10) |
| Cu-IUD | 89 (58.2) | 295 | 96.4 (85.7–108.1) | 1.38 (1.05–1.82) | 1.35 (1.03–1.76) |
| All LARCs | 839 (49.7) | 2,545 | 75.3 (72.4–78.3) | Not applicable | Not applicable |
| **Routine checkups at family planning clinics** |  |  |  |  |  |
| LNG-IUS | 467 (46.6) | 494 | 24.6 (22.5 t0 26.9) | Ref. | Ref. |
| Implant | 119 (22.3) | 129 | 12.1 (10.1–14.4) | 0.49 (0.40–0.60) | 0.41 (0.33–0.52) |
| Cu-IUD | 74 (48.4) | 79 | 25.8 (20.4–32.2) | 1.05 (0.83–1.33) | 1.00 (0.79–1.27) |
| All LARCs | 660 (39.1) | 702 | 20.8 (19.3–22.4) | Not applicable | Not applicable |
| **Other than routine check-up at family planning clinics** |  |  |  |  |  |
| LNG-IUS | 333 (33.2) | 606 | 30.2 (27.9–32.7) | Ref. | Ref. |
| Implant | 226 (42.4) | 444 | 41.7 (37.9–45.7) | 1.38 (1.15–1.65) | 1.10 (0.89–1.35) |
| Cu-IUD | 72 (47.1) | 149 | 48.7 (41.2–57.2) | 1.61 (1.22–2.12) | 1.48 (1.12–1.94) |
| All LARCs | 631 (37.4) | 1,199 | 35.5 (33.5–37.6) | Not applicable | Not applicable |

LARC, long-acting reversible contraception

CI, confidence interval

IRR, incidence rate ratio, calculated with negative binomial regression, adjusted with categorical age, history of pregnancy and history of sexually transmitted infection or visit for gynaecological reasons in primary or specialized health care or visit at the family planning clinics within the previous year

*In primary or specialized health care

**Supplementary Table 5. Visits in primary and specialized care for various gynecological reasons according to choice of LARC method.**

| **Reason for visiting** | **Total number of visits/diagnoses** | **Visits/diagnoses per 100 woman-years (95% CI)** |
| --- | --- | --- |
| **All gynecological reasons*** |  |  |
| LNG-IUS | 793 | 39.5 (36.8–42.4) |
| Implant | 407 | 38.2 (34.6–42.1) |
| Cu-IUD | 146 | 47.7 (40.3–56.1) |
| All LARCs | 1,346 | 39.8 (37.7–42.0) |
| **Menstrual problems*** |  |  |
| LNG-IUS | 244 | 12.2 (10.7–13.8) |
| Implant | 180 | 16.9 (14.5–19.5) |
| Cu-IUD | 41 | 13.4 (9.6–18.2) |
| All LARCs | 465 | 13.8 (12.5–15.1) |
| **Vaginal infections*** |  |  |
| LNG-IUS | 192 | 9.6 (8.3–11.0) |
| Implant | 74 | 6.9 (5.5–8.7) |
| Cu-IUD | 28 | 9.2 (6.1–13.2) |
| All LARCs | 294 | 8.7 (7.7–9.8) |
| **Visits for abortion care at family planning clinics or specialized health care** |  |  |
| LNG-IUS | 9 | 0.4 (0.2–0.9) |
| Implant | 18 | 1.7 (1.0–2.7) |
| Cu-IUD | 15 | 4.9 (2.7–8.1) |
| All LARCs | 42 | 1.2 (0.9–1.7) |
| **Diagnoses of STIs^†^** |  |  |
| LNG-IUS | 25 | 1.2 (0.8–1.8) |
| Implant | 21 | 2.0 (1.2–3.0) |
| Cu-IUD | 5 | 1.6 (0.5–3.8) |
| All LARCs | 51 | 1.5 (1.1–2.0) |
| **Pelvic inflammatory disease diagnoses*** |  |  |
| LNG-IUS | 54 | 2.7 (2.0–3.5) |
| Implant | 19 | 1.8 (1.1–2.8) |
| Cu-IUD | 14 | 4.6 (2.5–7.7) |
| All LARCs | 87 | 2.6 (2.1–3.2) |

LARC, long-acting reversible contraception

CI, confidence interval

STI, sexually transmitted infection

* In primary or specialized health care

^†^ According to the register of infectious diseases, including chlamydia, gonorrhea, and syphilis
